# Genetic Tools to Study Cardiovascular Biology

**DOI:** 10.3389/fphys.2020.01084

**Published:** 2020-09-24

**Authors:** Irene Garcia-Gonzalez, Severin Mühleder, Macarena Fernández-Chacón, Rui Benedito

**Affiliations:** Molecular Genetics of Angiogenesis Group, Centro Nacional de Investigaciones Cardiovasculares (CNIC), Madrid, Spain

**Keywords:** genetic tools, mosaics, cardiovascular, imaging, barcoding, transgenics, Cre, clonal analysis

## Abstract

Progress in biomedical science is tightly associated with the improvement of methods and genetic tools to manipulate and analyze gene function in mice, the most widely used model organism in biomedical research. The joint effort of numerous individual laboratories and consortiums has contributed to the creation of a large genetic resource that enables scientists to image cells, probe signaling pathways activities, or modify a gene function in any desired cell type or time point, à la carte. However, as these tools significantly increase in number and become more sophisticated, it is more difficult to keep track of each tool’s possibilities and understand their advantages and disadvantages. Knowing the best currently available genetic technology to answer a particular biological question is key to reach a higher standard in biomedical research. In this review, we list and discuss the main advantages and disadvantages of available mammalian genetic technology to analyze cardiovascular cell biology at higher cellular and molecular resolution. We start with the most simple and classical genetic approaches and end with the most advanced technology available to fluorescently label cells, conditionally target their genes, image their clonal expansion, and decode their lineages.

## Reporter and Cre Mouse Lines to Analyze and Genetically Target the Cardiovascular System

For many years, scientists relied on immunostainings to visualize the different cells composing the cardiovascular system. Nowadays, there are antibodies to specifically label most of the cardiovascular cell types. This technique allows the histological examination of an organ or tissue architecture, and the scoring of tissue malformations arising from genetic mutations. However, histological and immunostaining techniques are incompatible with live imaging and often do not allow the isolation of specific cell types. Given that most tissues are formed by tightly-associated cells, these techniques also do not allow the visualization of single cells within the tissue and the study of their morphology or dynamics.

**Figure 1:**
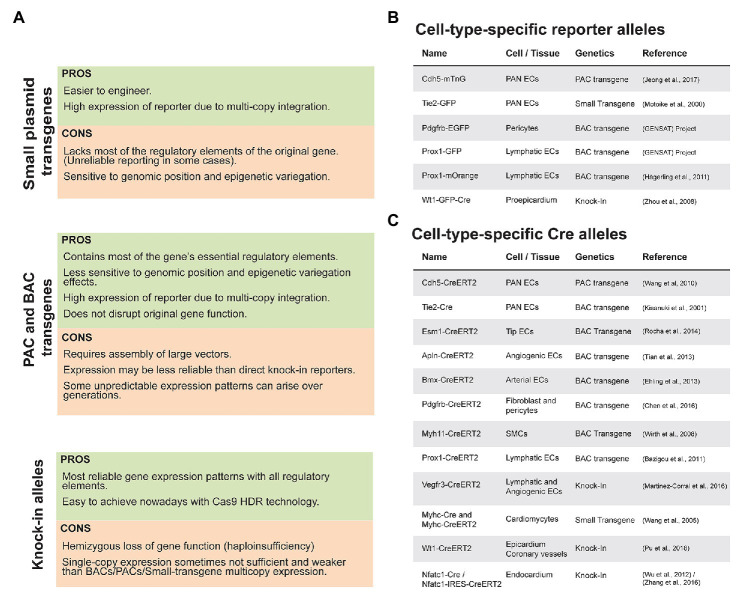
Pros and Cons of transgenic and knock-in lines used in the cardiovascular biology field. **(A)** Summary of advantages and disadvantages of different types of transgenic and knock-in alleles. **(B)** List of commonly used fluorescent reporter mouse lines in the cardiovascular biology field. **(C)** List of commonly used *Cre/CreERT2* mouse lines.

In order to live image and analyze cells at higher morphological and molecular resolution, scientists engineered new mouse models containing genes coding for fluorescent proteins (FP) or recombinases downstream of cell type-specific genes or promoters (Figure 1). There are many apparently redundant mouse lines available, using for example the commonly used *Tie2* and *Cdh5* promoters, but each transgenic line has its particularities given the type and location of the transgene. The first generation of mouse lines frequently contained multicopy insertion of small plasmid transgenes in the genome, which often lacked all the elements required to drive robust and specific expression of the FPs or recombinases in all desired cells. They were also sensitive to transgene and genomic position-related epigenetic variegation (Garrick et al., 1998; McBurney et al., 2002). These were followed by second generation mouse lines using larger transgenes such as bacteriophage P1-derived Artificial Chromosomes (PACs, up to 120 Kb) and Bacterial Artificial Chromosomes (BACs, up to 250 Kb) that can carry significantly larger DNA sequences containing most if not all of a gene essential promoter/enhancer elements. These larger transgenes were also significantly less sensitive to genomic position and epigenetic variegation effects (Giraldo and Montoliu, 2001; Adamson et al., 2011). Regardless of their size, transgenes expression is less reliable when compared with direct knock-ins of a reporter or recombinase gene in the native locus of the cell type-specific gene. There are many reports showing that unlike knock-ins, transgene expression can change throughout generations and result in highly unpredictable expression patterns (Koetsier et al., 1996; Mutskov and Felsenfeld, 2004). Knock-ins in the native locus usually guarantee stability and robustness in gene expression patterns. However, knock-in of a reporter within a gene was historically much more difficult to achieve, since it required assembly of large targeting vectors, their genome targeting in totipotent mouse embryonic stem (ES) cells and germline transmission to generate a genetically modified allele to the progeny (Westphal and Leder, 1997). However, with the advent of CRISPR/Cas9 technology, it is now possible to integrate by Cas9-induced DNA break and homology directed repair (HDR), small genetic cassettes downstream of virtually any mouse gene promoter. This is done by standard injection in mouse eggs of Cas9, a guide RNA and a donor DNA molecule containing homologous sequences flanking a DNA insert of interest (Yang et al., 2013; Platt et al., 2014; Chu et al., 2016; Scott and Gruzdev, 2019). This greatly eases the generation of gene or cell type-specific transgenic lines. Despite its current easiness, inserting a reporter or recombinase gene in-frame with the gene endogenous ATG has also disadvantages, such as the hemizygous loss of gene function. There are many reports showing a significant impact on cell biology of a 50% loss in gene expression, such as the haploinsufficiency of genes like *Vegfa*, *Dll4*, and *Kdr* (Carmeliet et al., 1996; Gale et al., 2004; Oladipupo et al., 2018). An alternative is to insert in the 3'-untranslated region (UTR) of a gene (Basak et al., 2018) an internal ribosome entry site (IRES) or a viral 2A peptide containing cassette (Trichas et al., 2008; Alvarez et al., 2015; Basak et al., 2018), in order to better preserve the targeted gene function. But as with everything, there are also cons of using these less disrupting strategies. Reporter genes when introduced downstream of IRES elements are less translated than the upstream genes (Al-Allaf et al., 2019), which may significantly decrease reporter expression and its detectability. In the case of the 2A peptide approach, care and pre-validation is required in order to avoid decreasing the function of the upstream protein, by the C-terminally fused 11 aa belonging to the 2A peptide. In addition, the 2A peptide decreases overall translation rates due to the required pause and ribosomal skipping step associated with the translation of the 2A-peptide-containing protein (Trichas et al., 2008; Sharma et al., 2012). Another important disadvantage of a gene knock-in is that it always results in single-copy expression, whereas a BAC or plasmid transgenic allele, particularly the best ones, usually contains multiple copies of the same transgene, which often results in higher reporter/Cre expression levels (Sörensen et al., 2009; Ubezio et al., 2016). A good example of this is the comparison between the BAC *Esm1-CreERT2* line (Rocha et al., 2014) that is highly expressed and induces the recombination of standard *Cre*-reporters in most retina endothelial tip cells, and the gene targeted *Esm1-H2B-Cerulean-2A-CreERT2* line (Pontes-Quero et al., 2019), which is significantly less expressed and recombines only few tip cells during retina vascular development.

Taken all these considerations in mind, the best strategy to follow in order to generate a good gene-reporter or gene-recombinase reporter line is dependent on the gene itself, its predicted loss-of-function phenotype, relative expression, and the availability of in house BAC transgenic or CRISPR/Cas9 technology. Use of small plasmid transgenesis or mouse ES cells gene targeting are clearly outdated technologies that on average result in either less robust results, or unnecessary delays and costs, respectively.

In Figure 1, we indicate some of the most important, validated and used mouse lines by the cardiovascular research community. There are many more available, but they are either less used or have significant disadvantages such as the nature of the transgene or reporter genes included. With the advent and ease of performing CRISPR knock-ins the trend is for more and better knock-in lines to appear, but as mentioned above, they have significant disadvantages in relation to BAC transgenic lines. What is so far a largely unappreciated and unused technology is the direct CRISPR/Cas9 targeting and modification in mouse eggs of existing and validated BAC transgenic lines, to create new alleles driving the stronger expression of the latest generation and improved versions of fluorescent reporters or recombinases.

**Figure 2:**
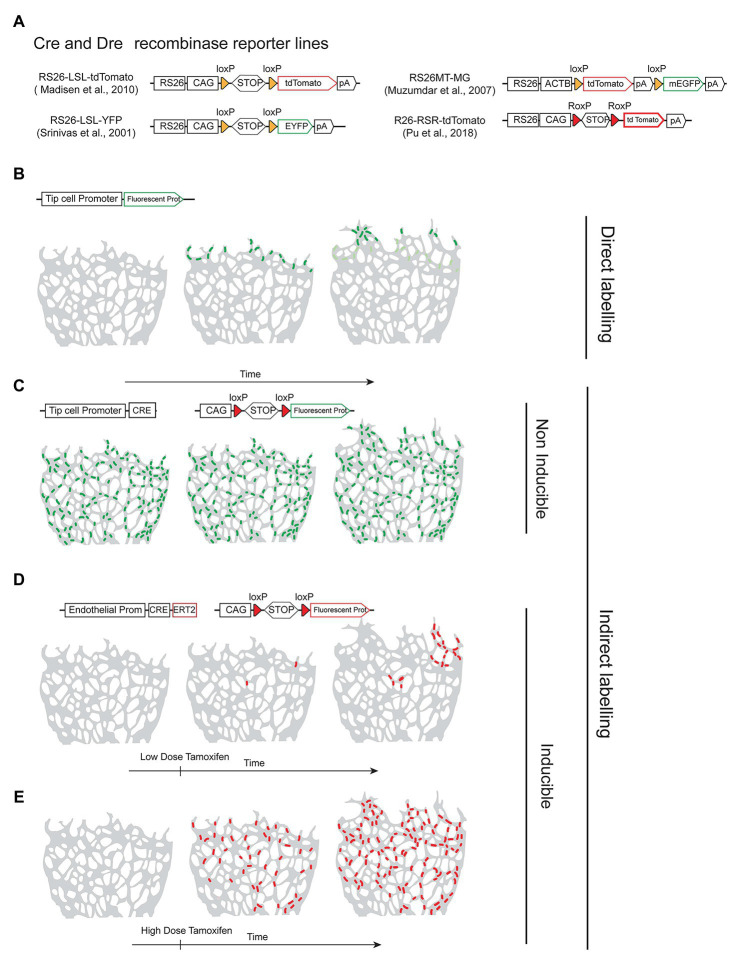
Recombinase reporter lines and their outcomes. **(A)** Schematic overview of distinct recombinase-inducible and Rosa26-locus targeted fluorescent reporters. **(B)** Example of direct labelling achieved by using an allele containing a vascular tip-cell-specific promoter driving expression of a fluorescent protein in the retina angiogenesis model. Only tip cells express the promoter and the reporter protein. **(C)** Example of a non-inducible and indirect labelling strategy using a tip-cell-specific *Cre* line in combination with a fluorescent reporter line. Cre is expressed in tip-cells and all their progeny during tissue development and therefore the fluorescent reporter is expressed in many endothelial cells, not only tip cells. **(D,E)** Example of indirect labelling using an inducible *CreERT2* line expressed in all endothelial cells, in combination with a reporter line. Cre is only activated after tamoxifen administration and recombination/cell labelling occurs at a controlled time-point. Number of recombination events and cellular labelling can be modulated by the dose of tamoxifen.

To label a given cell of interest, one can use a cell-specific-gene reporter mouse line that allows the direct labeling of cells expressing a given gene (Figure 1B) or an indirect labeling strategy combining a specific-gene *Cre* line (Figure 1C) with an ubiquitous and Cre-inducible reporter (Figure 2A). The first strategy has the advantage of allowing the direct labeling of cells expressing a given gene and hence it reports also the dynamics of the selected gene expression. However, it has the disadvantage of the selected gene-promoter elements not driving sufficiently high levels of the reporter, or the gene may be downregulated and no longer labels the intended cells once a given biological process is completed. An example is the expression of the tip-cell enriched gene *Esm1* (Figure 2B) or the angiogenesis restricted gene *Apln*. These genes are not expressed in more mature and quiescent vessels. The strategy involving a combination of an allele driving the expression of the recombinase and a reporter allele has the advantage of allowing the permanent labeling of cells with a constitutively expressed fluorescent reporter, usually driven by the very strong and ubiquituous *CAG/ROSA26* promoters. This usually results in much higher levels of FP expression, which facilitates its detection and increases the cellular resolution in sections or large volumes of tissue. With this strategy, all the progeny of the initially labeled or recombined cells will be labeled, which may lead to lack of specificity in the labeling (Figure 2C). One example is the frequent use of the *Tie2-Cre* or *Cdh5-Cre* expressing lines to label endothelial cells. Since embryonic endothelial cells can differentiate to hematopoietic progenitor cells (Ottersbach, 2019) this results in the labeling of the entire hematopoietic compartment as well, which may complicate the imaging of endothelial cells, particularly in inflammation settings, and also confound the interpretation of a given genetic lineage tracing experiment (Alva et al., 2006). In addition, gene-specific *Cre* lines may be weakly expressed in many other cell types, and trigger the recombination of the reporter allele and labeling of cells that are not usually considered to express the gene of interest, a phenomenon usually named as Cre leakiness and a frequent source of lineage-tracing artifacts and misinterpretations. Therefore, standard *Cre* lines should not be used for lineage tracing experiments. Instead, *CreERT2* lines should be used, since they provide temporal control over a genetic modification and are less capable of inducing non-intended recombination of a floxed allele in cells weakly expressing a *CreERT2* transgene. *CreERT2* lines also enable single-cell clonal analysis at low doses of tamoxifen (Figures 2D,E).

## Using Recombinase Technology to Study Cardiovascular Gene Function: Mind Your Cre, Floxed Gene, and Reporter Alleles

The discovery of recombinases that are able to recognize unique palindromic DNA sequences not present in the mammalian genome, and that promote their recombination (deletion/inversion), paved the way to recombinase-based conditional genetic gain and loss-of-function technology (Branda and Dymecki, 2004; Birling et al., 2009). So far the most potent recombinase at 37°C and in mammalian cells is Cre (Anastassiadis et al., 2009), that is able to recognize 34 bp DNA sequences named as *LoxP*. This prompted the generation of thousands of mouse lines containing genes flanked by *LoxP* sites (floxed), and transgenes driving expression of Cre/CreERT2 in specific cell types (Murray et al., 2012). The Cre/*lox* technology requires the combination in the same mouse of one allele driving the expression of Cre or CreERT2 in a particular cell/tissue/organ and floxed alleles. This then enables a given floxed allele to be deleted or activated conditionally to the expression of Cre or its activation by tamoxifen (CreERT2). The final outcome of the technology is the ability to control genetic modifications in space and time.

Recombinase technology can be used not only to label and fatemap cells or tissues of interest (Figure 2) but also to perform inducible or conditional genetic deletions. The ability to delete a given known floxed gene at any given time and in any cell/tissue has revolutionized biomedical science and particularly the study of cardiovascular development. It is well-established that gene function varies over time and the cell where it is expressed. It is also known that organs across our body communicate or cross-talk with each other. Therefore, the information arising from the analysis of embryos or animals with a germline deletion of a given gene is often incomplete and its tissue-specific or primary function may be difficult to assess or understand. The global knockout (KO) of genes relevant for cardiovascular development or basic cellular functions, such as cell proliferation or differentiation are also frequently embryonically lethal, impairing the study of a gene function in subsequent biological processes or in adult organisms. Although it is still common practice in biomedical science, germline gene deletion or in a tissue-specific manner from early embryonic development should be avoided if the purpose is to understand its function in postnatal or adult cancer/heart biology. This is particularly relevant in studies conducted to assess if targeting a given gene/pathway is relevant for the progression of an adult organism disease. Gene function in the adult organism or disease can only be safely assessed by genetically targeting the adult tissue after the onset of the disease, something too often ignored by the biomedical community.

Despite the significant advantages of using conditional genetics, it is often significantly more difficult to achieve the desired conditional genetic modification. In the case of a germline KO mouse line, once it is established, no further gene loss-of-function validation is required. However, in conditional genetics, each and every single experiment needs its proper controls of gene deletion and numerous studies have demonstrated the need for caution in the use of conditional genetic technology. The main problem is that it is often not possible to control all aspects of a conditional genetic experiment. There are three main issues or caveats associated with recombinase-based conditional genetics. The first is related with the transgenes used to drive expression of Cre/CreERT2. There are very often significant and unpredictable position-effect variegation of transgenes due to random epigenetic silencing, which is so far impossible to predict or control. This can lead to variability in Cre/CreERT2 expression within cells of a tissue, littermates, or across generations of mouse breedings (Garrick et al., 1998; Long and Rossi, 2009), turning conditional genetics unpredictable and often unreliable and costly. Even assuming an invariable and high expression of the *Cre/CreERT2* allele in your cells/tissue/organ of interest, the second issue with *Cre/Lox* technology is that *Cre* recombination depends on the location of the *LoxP* sites in the genome and the genetic distance and DNA sequence separating them. Therefore, for every floxed allele one should expect a different recombination efficiency, even when using the same *Cre* or *CreERT2*-expressing allele. An example of how this can affect interpretation of results is a combination of studies in the cardiovascular biology field showing that previously reported cardiac fate mapping studies were misguided by relatively low recombination efficiencies of the reporters used. *Isl1-cre* and *Nkx2-5-Cre* lines were previously used to label and follow distinct cardiac progenitors, using a *CMV β-actin-nlacZ* and a *RS26-β-galactosidase* reporter, respectively. Since in these first studies cells recombined with *Isl1-Cre* line were found to contribute mainly to the second heart field (SHF: right ventricle, outflow tract and atria; Cai et al., 2003), and cells recombined with *Nkx2-5-Cre* were found to give rise mainly to cardiomyocytes (Moses et al., 2001) the authors of these two studies concluded that these cells arise from distinct progenitors. However, in a more recent study, in which the authors used the same Cre lines, but instead used the *Gata4-flap* reporter, significantly more cells were recombined and *Isl1* expressing progenitors were found to contribute not only to the SHF but also to the first heart field (FHF: left ventricle). Similarly, Nkx2-5 progenitors were found to give rise, apart from cardiomyocytes, to endothelium and smooth muscle cells (Ma et al., 2008). In addition to this variability in sensitivity to Cre of distinct reporter alleles located in different loci, recent studies have compared and found significantly different recombination efficiencies of several distinct floxed reporter alleles located in the same *Rosa26* locus (Vooijs et al., 2001; Pontes-Quero et al., 2017). These differences are clearly not only related with the genetic distance between the *LoxP* sites or their position in the genome, but also the nature of the DNA sequences surrounding the *LoxP* sites. This brings us to the third and often overlooked caveat of conditional genetics, which is the reliance on independent fluorescent reporters of recombination to validate the conditional technique and analyze cells having the intended specific genetic deletion. These recombinase reporters are usually alleles targeted to the ubiquitous mouse *Rosa26* locus (Soriano, 1999; Srinivas et al., 2001) and are activated or expressed only after the cell expresses Cre or has induced CreERT2 activity (Figure 2A). However, since there is no genetic linkage between the reporter allele and any other floxed alleles in the cell, the correlation between the recombination of a reporter allele and another floxed allele is often very low. Indeed a recent study showed that some commonly used *Rosa26* reporter alleles overreport genetic deletions, whereas others underreport (Fernández-Chacón et al., 2019). In our hands, the commonly used *R26-LSL-YFP* allele (Srinivas et al., 2001), and particularly the *R26-LSL-tdTomato* reporter allele (Madisen et al., 2010), are extremely sensitive to Cre/CreERT2 activity, unlike the majority of other tested floxed genes (Fernández-Chacón et al., 2019). Therefore, when using CreERT2, having a tissue full of cells expressing a recombinase reporter, does not guarantee that your gene is deleted.

For all conditional genetic experiments, it is therefore critical to have reliable methods to confirm that a given gene (not the reporter) is properly recombined/deleted, and only in the desired cell type. However, the large majority of studies employing conditional genetics rely on the use of fast PCR-based or Western blot-based methods to confirm genetic deletions in whole tissues or groups of isolated cells. These methods are insufficient because they only indicate the average gene-deletion efficiency, and cannot quantify the heterogeneity in genetic deletion efficiency among different cells of different tissues, which can lead to misinterpretations of a given conditional mutant phenotype. Also these methods are performed with cells or tissues that were lysed for DNA/Protein extraction, and therefore the tissues/cells analyzed for efficiency of genetic deletion are not the same as the ones used for microscopy/phenotypic analysis. The gold standard and safest method for confirming inducible and specific gene deletion is to co-immunostain for the encoded protein and another tissue or cell marker in the same tissue. However, for most proteins there are no high-quality antibodies able to distinguish between the morphology of cells with and without protein expression in the tissue. Another issue is that gene transcription and protein stability oscillate in a cell, and thus a cell with no detectable expression of a given gene or protein at a given moment may still be wildtype for the coding gene.

**Figure 3:**
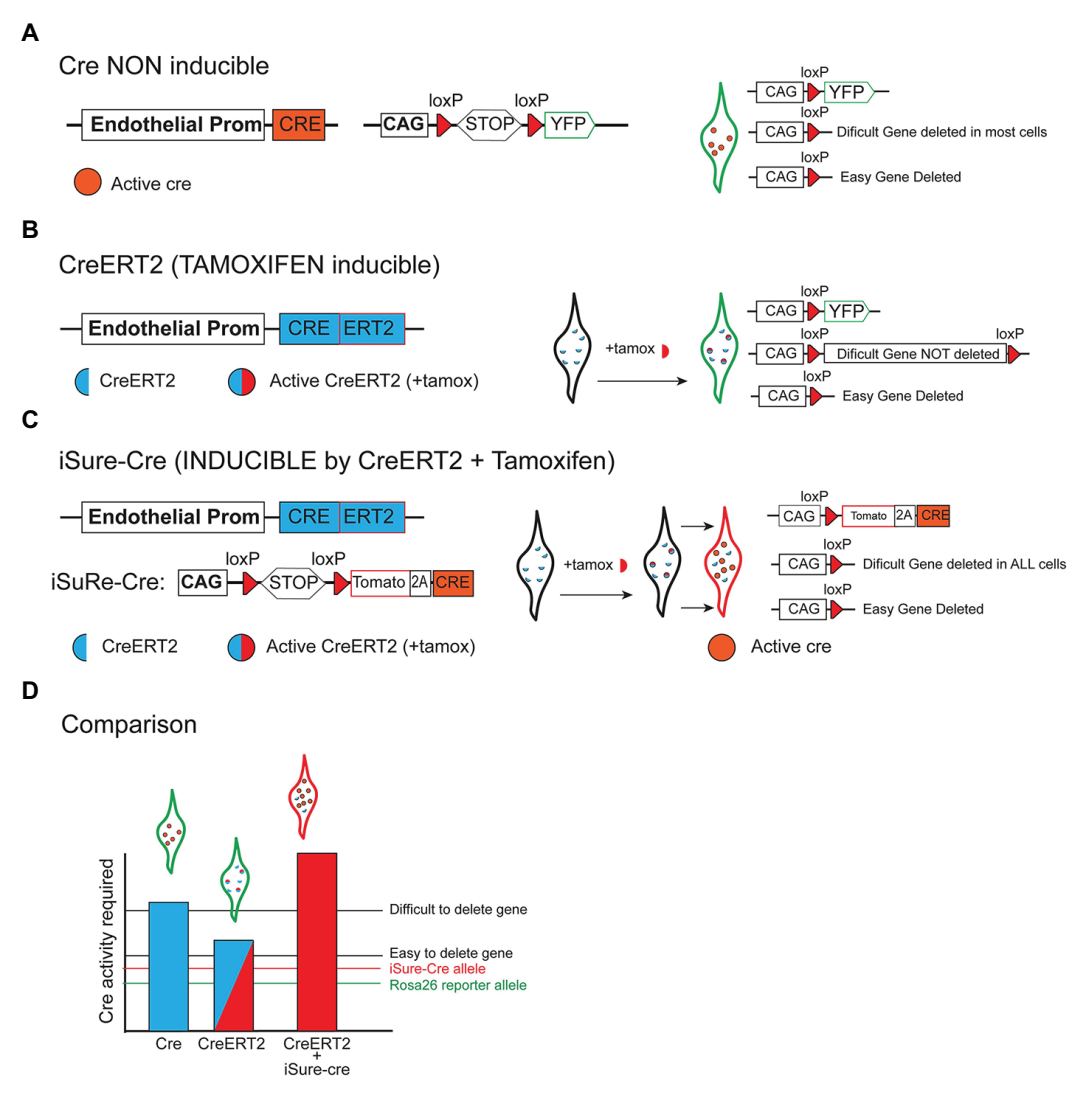
Different strategies to delete floxed genes. **(A)** Scheme showing that when a constitutively active Cre allele (non inducible) is combined with a reporter allele, most cells expressing the reporter recombine/delete both easy and difficult floxed genes. **(B)** When tamoxifen-inducible CreERT2 alleles are used, many cells expressing the Cre-reporter allele may not delete other genes that are more difficult to delete since these require a higher or sustained level of recombinase activity. **(C)** The iSure-Cre allele is inducible by CreERT2, and enables a higher Cre activity in reporter-expressing cells. The use of this strategy guarantees full deletion of floxed genes in reporter-expressing cells. **(D)** Illustrative chart comparing the genetic strategies mentioned above. The bars represent the Cre activity achieved with most Cre/CreERT2 lines and the iSuRe-Cre allele. Different floxed genes require a different level of Cre activity to be deleted (an easy and difficult to delete gene is represented). Only the iSuRe-Cre allele can ensure the deletion of most genes in reporter-expressing cells.

The caveats of conditional genetics mentioned above are significantly more apparent and pronounced when using tamoxifen inducible-*CreERT2* lines, or weaker recombinases such as Dre and Flp. In these experiments, the level of recombinase/Cre activity is significantly lower, and often it is not sufficient to delete two copies of a floxed gene, even though it may be sufficient to recombine and activate a single and sensitive recombinase-reporter allele located in the accessible *Rosa26* locus (Compare Figure 3A with Figure 3B). This is even more relevant when incomplete genetic induction schemes are used, such as when the induction of low frequency genetic mosaics is desired in order to track single-cell-derived clones or lineages (Figure 2D). In this case, most cells expressing the recombinase will not be recombined, and therefore the assumption that the few cells showing recombination of a given reporter allele, have also recombined the intended floxed gene, is even more incorrect.

To overcome the above-mentioned problems associated with conditional genetic deletions, we recently generated a new inducible dual reporter-Cre mouse allele (Figure 3C), named as iSuRe-Cre (Fernández-Chacón et al., 2019). This allele enables the significant increase of Cre activity in reporter-expressing cells, providing certainty that these cells have completely recombined all floxed alleles, regardless of their sequence and location in the genome (Figure 3D). This tool greatly decreases the occurrence of false positives, i.e., cells expressing a fluorescent reporter and not having other gene/s fully deleted. The tool is particularly relevant for epistasis studies, in which multiple genetic deletions are needed in the same cell or tissue. With the iSuRe-Cre allele, because reporter-expressing cells express also Cre and not only the reporter, efficient genetic deletions can also be achieved in a mosaic fashion. In this way, we can have single cells reliably reporting gene deletion within a wildtype tissue, even if the initial Cre/CreERT2-dependent recombination induction was very low. This tool also enables direct conditional genetics and live imaging experiments, since reporter-expressing cells are certainly mutant. In the past, this was always challenging and involved independent and very time-consuming experimental controls, because it is not possible to validate genetic deletions in live cells, given the need to fix or isolate them before performing the necessary genetic deletion validation experiments. In summary, this genetic tool increases the ease, efficiency, and reliability of conditional genetic experiments.

An alternative method presented recently to increase Cre activity after tamoxifen induction of CreERT uses an inducible self-cleaved CreER (sCreER), in which the ER element is flanked by *LoxP* sites. In the non-induced state, Cre-LoxP-ER-LoxP is expressed but inactive, and after tamoxifen the ER portion is deleted in the induced cells, and Cre is free to mobilize to the nucleus and recombine other flanked genes (Tian et al., 2020). This strategy boosts Cre expression and activity in initially CreER recombined cells; however, the sCreER lines generated so far are tissue-specific and also do not contain an internal reporter linked with Cre expression, in order to ensure that cells expressing an independent Cre reporter have full gene deletion.

## Using Intersection Genetics for Higher Genetic Resolution

So far, we have mentioned the most common techniques and approaches used to label specific tissues and cell types in order to distinguish or isolate them for downstream analysis, or perform tissue-specific genetic modifications. However, sometimes a single gene promoter is not sufficient to drive the expression of a reporter or Cre to a specific tissue, or fraction of a tissue, of interest. Intersection genetics in mice was made possible after the discovery of other recombinases such as Flp and Dre (Anastassiadis et al., 2009). Intersection genetics relies on the sequential or simultaneous intersection in time or space of two different recombinase systems, so that one is conditional or dependent on the other (Rodríguez et al., 2000; Anastassiadis et al., 2009). This leads to cascades of recombination and the differential labeling or targeting of specific cell populations (Dymecki et al., 2010; Plummer et al., 2015).

**Figure 4:**
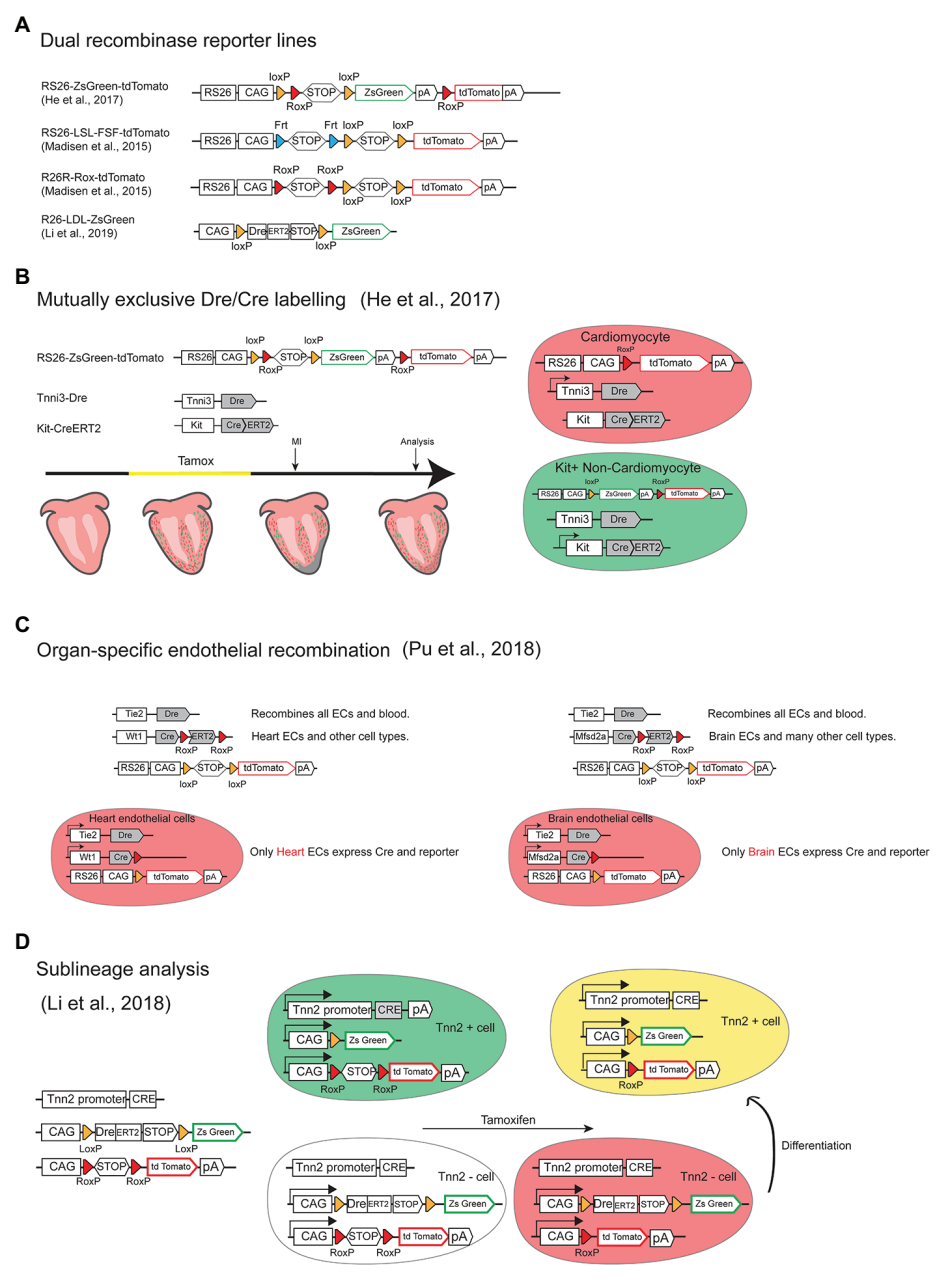
Intersection genetics by combining Cre and Dre mouse lines. **(A)** Schemes of the alleles of dual (Cre/Dre or Cre/Flp) reporter lines available for intersection genetics. **(B)** Scheme of the alleles and strategy used to achieve mutually exclusive Cre and Dre recombination and labeling of cardiomyocyte (Tnni3+/tomato+) and non-cardiomyocyteckit+ cells (Tnni3-/ZsGreen+) to enhance precision of lineage tracing after administration of tamoxifen and myocardial infarction (MI). **(C)** Scheme of the alleles and strategy used to achieve organ-specific (heart or brain) endothelial recombination. **(D)** Scheme of the alleles and strategy used to perform sublineage and fate-mapping analysis of Tnn2+ and Tnn2- cells differentiation to cardiomyocytes.

Several Dre and Cre mouse lines and dual reporters were generated to perform dual labeling and intersection genetics in the cardiovascular system (Figure 4A). In one study, the Bin Zhou laboratory used dual-recombinase-activated lineage tracing with interleaved reporter (DeaLT-IR) to significantly enhance the precision and reduce unintended Cre-LoxP lineage tracing (Figure 4B). Using DeaLT-IR they could distinguish cardiomyocytes (Tnni3+/tomato+) from non-cardiomyocytes Kit+ cells (Tnni3−/ZsGreen+) to clearly show that Kit+ non-cardiomyocytes do not give rise to cardiomyocytes in the injured heart and that there is no contribution of Sox9+ biliary epithelial cells to hepatocytes in the liver after injury, two findings against previous dogma arising from the use of simpler or lower resolution genetic approaches (He et al., 2017).

The same laboratory also used similar intersection genetics to solve a recurrent problem in the vascular research field, which is that most endothelial-specific genetic modifications target the entire endothelium of all organs, and not specific organ vascular beds, which may lead to early lethality or distort the interpretation of a gene function in a particular vascular bed. In a very elegant study, Pu and colleagues generated a set of genetic tools that allow the specific targeting of heart or brain endothelium, and not of other organs vessels (Pu et al., 2018). The alleles generated allow the expression of active Cre, and therefore genetic deletions, specifically in Tie2+/Wt1+ coronary ECs or Tie2+/Mfsd2a+ brain ECs and not other organ vascular beds (Figure 4C). Using this approach they could delete the most important VEGF receptor (Vegfr2) in each endothelial compartment and understand its organ-specific endothelial function circumventing early lethality and phenotypes derived from deletion in other organs. Thanks to this tool they discovered that Vegfr2 is essential for the integrity of the blood brain barrier (BBB) and for angiogenesis in the central nervous system.

In another study, they used similar mutually exclusive dual recombinase systems (Figure 4D) to investigate if all non-cardiomyocyte cells, can originate cardiomyocytes at distinct stages of development and adult tissues, a long-standing question in the field. Using this genetic technology they found that non-cardiomyocytes can only differentiate to cardiomyocytes before E11.5, not at later stages or during neonatal heart regeneration (Li et al., 2018).

## Multispectral Fluorescent Mosaics for High Resolution Single-Cell or Clonal Analysis

The above-mentioned lines are sufficient to distinguish cell types and decipher gene function at the tissue or organ level. However, higher genetic and cellular resolution is often required to understand single-cell biology, particularly when live cell imaging is not possible. Tissues are formed by the association of highly heterogeneous cells, each having different proliferation, differentiation or migration properties. Standard conditional genetics is convenient to analyze the average phenotype of wildtype or mutant cell populations within a tissue, but not to discern single-cell biology.

For long, scientists have tried to track and follow single cells from the initial stages of embryonic development to their final stages of differentiation. *Drosophila melanogaster* or *Caenorhabditis elegans* development was initially mapped by visual tracking of non-fluorescent cells, where the researchers had to record the development of the early embryos over time (Sulston and Horvitz, 1977; Underwood et al., 1980; Sulston et al., 1983). With the advent of transgenics expressing FPs, live imaging of tissues, or individually labeled cells became possible, particularly in lower vertebrate organisms, such as zebrafish (Pan et al., 2013). In the mouse, however, it is still not possible to live image most cell types and tissues, particularly for long periods of time. The discovery of inducible site-specific recombinases (i.e., CreERT2, FlpOERT2, or DreERT2) was therefore an important breakthrough to study single-cell biology since they enabled the pulse and chase of recombinase reporter-expressing cells, or single-cell fate mapping experiments (Legue and Joyner, 2010). This allowed the precise induction of permanent genetic modifications only in a few cells within a tissue (with a low dose of inducer/tamoxifen), and since these are inherited by all the daughter cells, it is possible to perform restrospective clonal analysis to its ancestor (Figure 2D). This technique allows the quantification of how a given single progenitor cell proliferates, differentiates or migrates over time in the tissue. However, depending on the experimental window and dynamics of the tissue, in order for this simple pulse-and-chase restrospective clonal analysis method to be accurate, only very few cells in a tissue or embryo can be induced or recombined, otherwise there is a loss of single-cell or clonal resolution (Compare Figure 2D vs. Figure 2E). Since with this technique only very few cells, or clones of cells, can be pulsed and reliably followed over time, this method has a very high cost given the large amount of embryos/animals and time needed to generate and analyze enough single-cell-derived clones of cells. This methodological bottleneck, associated with the development of new FPs and multispectral confocal microscopy, created the need to engineer new DNA constructs and mouse lines that allow the induction and detection of numerous clones of differentially labeled cells in the same tissue.

The first mouse line to be developed in order to effectively achieve differentially labeling of multiple cell clones was named as *Brainbow*, and it enabled combinatorial multispectral mosaics in the brain of mice, given the use of the *Thy1* promoter (expressed in brain cells). This method was based on the existence of mutually exclusive Lox sites (*LoxP*, *LoxN*, and *Lox 2272*) that after CreERT2 induction, can be recombined in only one of three possible ways, resulting in the expression of only one of three possible FPs (RFP, YFP, CFP) in each cell (Livet et al., 2007). Since the *Brainbow* transgenic alleles contained the tandem integration of 8–16 copies of a single three-Loxed/FPs construct, they enabled the induction and distinction of up to 90 different combinations of FPs/clones in the same tissue. Given the numerous possibilities and diversity of the stochastic recombination event, this method allowed the safe labeling and distinction of multiple single-cells and their progenies in the brain.

**Figure 5:**
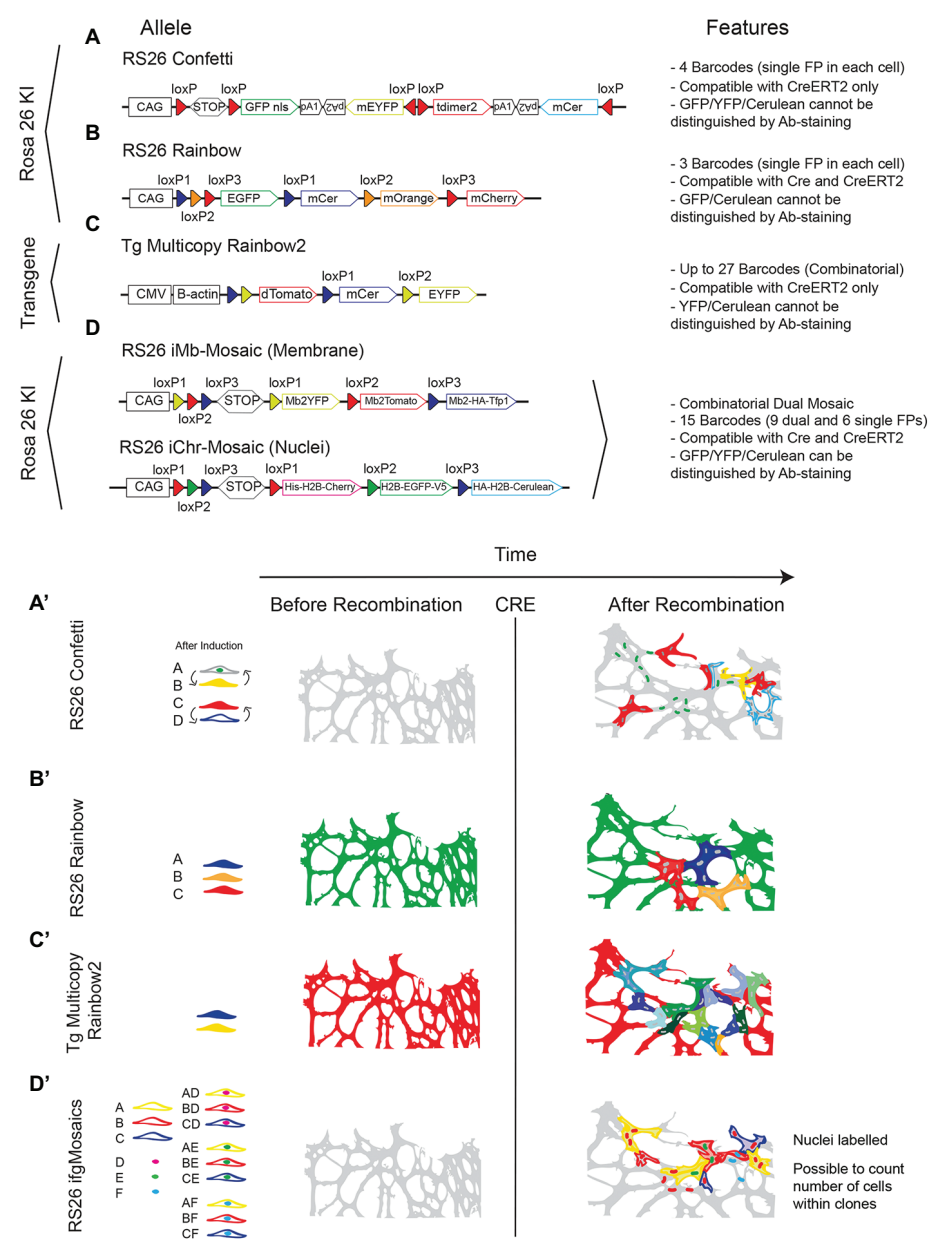
Multispectral fluorescent mosaic alleles. **(A-D)** Schemes of the alleles and main features of available mouse lines to induce stochastic fluorescent genetic mosaics in all cell types. **(A’-D’)** Illustration showing the comparison of the endothelial cell labelling achieved before and after Cre activity. Note the difference in colors/channels and cellular/clonal resolution.

However, the most widely used multispectral mouse line is not the *Brainbow*, but the *Confetti* (Snippert et al., 2010). In contrast to the *Brainbow* mice, the *Confetti* mouse carries a single brainbow construct (*Brainbow 2.1*) downstream of the strong and ubiquitously expressed CAG promoter and is inserted in the safe and ubiquitously expressed *Rosa26* locus, and therefore can be induced in any cell type. This mouse allows the labeling of individual cells with only one of four different FPs (nGFP, mEYFP, tDimer2, and mCerulean; Figures 5A,A’). This is a clear disadvantage when compared with the *Brainbow* combinatorial and multicopy alleles that allow significantly more diverse labeling. However, it provides for greater clarity in clone labeling and identification, since the separation of the different relative intensities and nuances of the three channels in *Brainbow* mice can often be challenging, particularly for beginners in microscopy imaging or image analysis.

There are now many derivations of the ubiquituous *Confetti* line and the main differences among them are the targeting or not to the *Rosa26* locus, the relative position of the cassettes, FPs of choice and compatibility or not with standard Cre (Figures 5B,B’,C,C’).

An alternative to the use of the *Confetti* or *Rosa26* Rainbow mice, is the use of the new *Dual ifgMosaic* mice (Pontes-Quero et al., 2017), with which it is possible to induce in any cell type, up to 15 (instead of four) different clones of cells, six expressing a single FP and nine expressing two FPs, one localized in the nuclei or chromatin (H2B tag) and the other in the membrane (Figures 5D,D’). The *Dual ifgMosaic* mouse lines also have the advantage of allowing distinction of the 15 clones of cells not only by direct live imaging of the spectrally distinct FPs, but also by immunostaining, since the different FPs used (MbYFP, MbTomato, HA-MbTfp1, His-H2B-Cherry, H2B-GFP-V5, and HA-H2B-Cerulean) contain distinct tags (HA, V5, or His) that enable distinction of the 15 clones of color-codes by only three antibody signals (Anti-GFP-488, Anti-Dsred-546, or Anti-HA-647). The *Confetti* and *Brainbow* mice rely on the multispectral detection of YFP/GFP/Cerulean, that are not distinguishable by antibody staining and do not allow the simultaneous distinction of the nuclei (for cell unit count) and membrane/filopodia (for cell shape). This is particularly important to quantify the number of cells in clones of epithelial cells (such as endothelial cells) that usually are tightly adherent to each other and are therefore non-distinguishable if labeled with only one cytoplasmic or membrane FP (Figures 5A’–D’). Another advantage of *Dual ifgMosaic* mice is the compatibility with Cre (not only CreERT2) expressing mouse lines. In these mice, each recombination event is based on permanent genetic deletions, which are induced when *LoxP* sites are positioned in the same orientation, whereas with *Confetti* the recombination event is based on a reversible genetic inversion that occurs when the LoxP sites are positioned in an opposite orientation (Nagy, 2000; Compare Figure 5A with Figure 5D). Moreover, in multi-copy tandem transgenic lines like *Brainbow* or the *Rainbow2* (Figure 5C), constitutive Cre expression leads to the deletion of the entire multicopy transgene and the full loss of clonal diversity, rendering them incompatible with standard Cre lines or the new iSuRe-Cre line (Fernández-Chacón et al., 2019).

## Reliable Functional Genetic Mosaic Systems

Functional genetic mosaics are very useful to understand the cell-autonomous role of a given gene. Most studies taking conclusions about a given gene cell-autonomous role, obtain it using standard conditional tissue genetics, and not single-cell genetics. When a gene has a severe impact on cardiovascular development or function, it is difficult to really analyze and understand its cell-autonomous role, given that the surrounding embryonic tissues or organs also do not develop or function well, which feedbacks and induces changes in the biology of the cardiovascular system. These inter-tissue cross-talks and feedbacks prevent the understanding of the real cell-autonomous gene function. An example of this is the consequence of deleting the gene *Dll4* in vascular cells during embryonic development. It leads to early embryonic death and significantly less vascular development, even though the gene is a suppressor of angiogenesis (Duarte et al., 2004; Suchting et al., 2007; Pontes-Quero et al., 2019). Inducible functional genetic mosaics on the other hand, allow us to study the cell-autonomous gene or pathway function for longer periods of time, without the confounding effects from the changing surrounding environment, given that non-mutant (generally named as wildtype) and mutant cells are located in the same tissue microenvironment.

We have mentioned above the caveats of using independent reporters of Cre activity, to induce the mosaic labeling of the desired mutant cells, given the low correlation between recombination of a reporter allele and another floxed gene of interest, particularly with *CreERT2* alleles and tamoxifen. We also showed how the new *iSuRe-Cre* allele (Fernández-Chacón et al., 2019) can be used to ensure genetic deletions in reporter-expressing cells. However, like for any other standard reporter allele, the *iSuRe-Cre* allele does not prevent the occurrence of false negatives, since cells not expressing or recombining the reporter allele may recombine other floxed alleles/genes that are more sensitive to Cre/CreERT2 activity. Therefore, none of these reporter alleles should be used to induce and analyze genetic mosaics of non-mutant and mutant cells, because they do not allow the labeling of the non-mutant cells, and most (with the exception of the *iSuRe-Cre* allele) do not even reliably report mutant cells.

**Figure 6:**
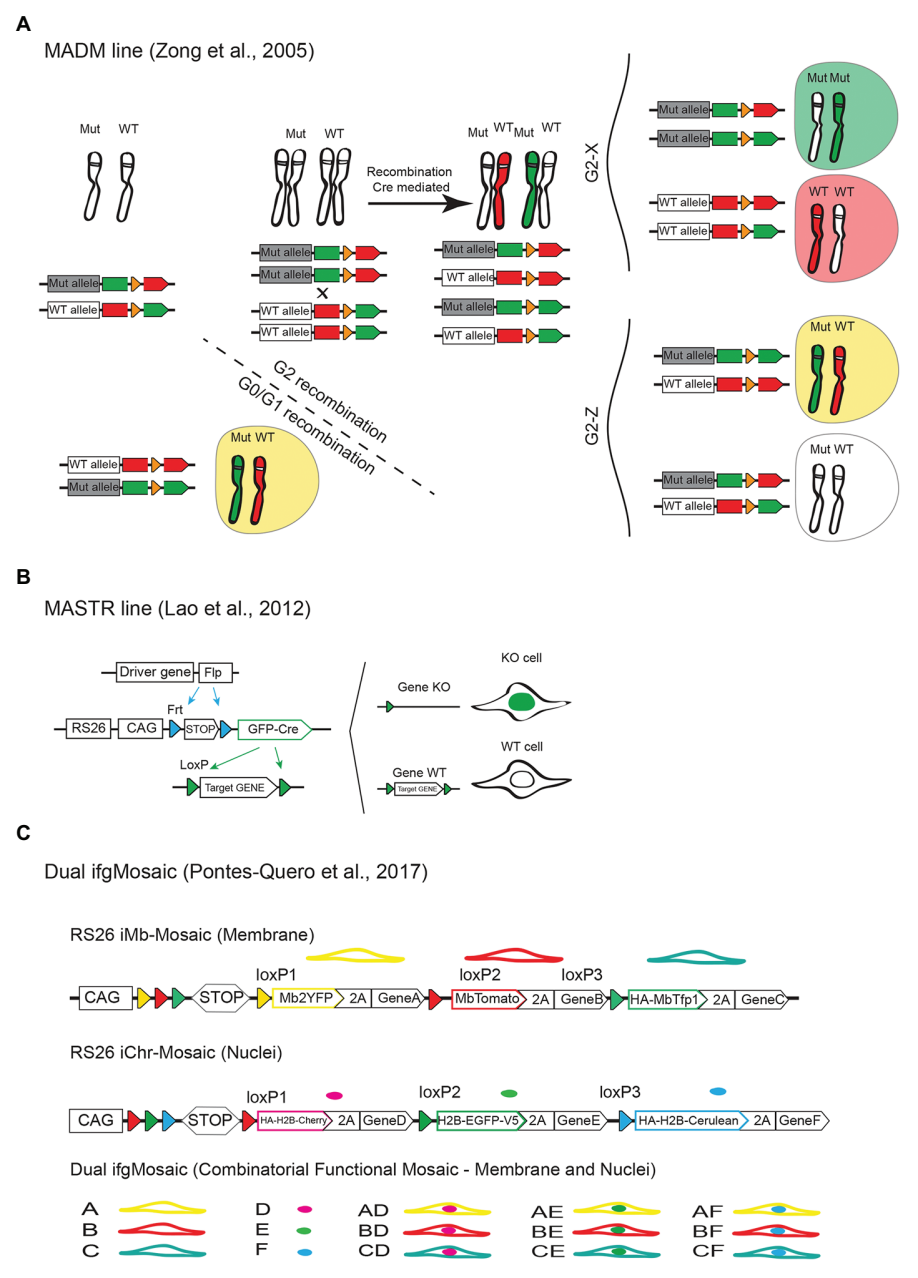
Mouse alleles for functional genetic mosaics. **(A)** The MADM approach is dependent on rare interchromosomal and Cre-dependent recombination events between wildtype and mutant/KO alleles linked to the expression of different fluorescent proteins. This results in the labeling of wildtype (Wt) and mutant (Mut, homozygous or heterozygous) cells with different fluorescent proteins/colors. **(B)** Scheme of the MASTR mouse line and how it allows the Flp-recombinase dependent labelling of the nuclei of cells expressing GFP-Cre and therefore having a given floxed gene deleted (KO). Note that with this approach wildtype cells are not labelled. **(C)** Dual ifgMosaic alleles enable multispectral and combinatorial gene expression for accurate mosaic/clonal functional genetics.

For reliable functional genetic mosaics, there must exist a complete (or almost complete) correlation between expression of a given marker/reporter and the genetic status of the cell, so that there is certainty that the cell in question carries the desired genetic modification, without having to worry about false positives or false negatives. The first system showing that it is possible to genetically link a cell reporter expression with the reliable and accurate labeling of mutant and wildtype cells in a mouse tissue was the mosaic analysis with double markers (MADM) system developed by the Liqun Luo laboratory (Zong et al., 2005; Zong, 2014). The MADM approach (Figure 6A) is based on the same principle of *Drosophila* mitotic genetic mosaics. It relies on interchromosomal recombination events between a mutant allele and wildtype allele, which are linked to reporter proteins, to generate differentially labeled control and mutant cells. This system is very useful to study the cell-autonomous effect of a given mutation since the induced labels for wildtype and mutant cells arise from the same progenitor cells and will have the same genetic background and biological context. This technology was used to study the cell-autonomous role of p27 in endothelial cells and its function in cell competition. Interestingly the authors found that in global *p27^KO^* animals, *p27^KO^* cells proliferate only 0.6 fold more than wildtype cells, whereas when using MADM mosaic animals, and the *p27^KO^* cells are surrounded by wildtype cells, they proliferate up to six times more (Defoe et al., 2020). This and other studies using genetic mosaics (Claveria et al., 2013) show how important are these functional mosaic methods to understand cell biology. They may not be relevant to understand the consequences of germline mutations or pharmacological treatments in disease, but they are very important to understand the origin and development of mosaic, or somatic mutations that are at the origin of most cancers and vascular malformations (Fernandez et al., 2016). Despite its great utility, the MADM approach has several drawbacks. The first is related with the very rare frequency of the Cre-dependent interchromosomal recombination events. Even when using constitutive Cre-expressing mouse lines, only very few clones of labeled mutant and wildtype cells are generated in the entire embryo. For this reason, there is no control over the timing of the recombination events and very few clones are obtained for statistical analysis. In addition, the requirement for genetic linkage between the engineered MADM elements and another gene mutation, limits its broad utility, as two *MADM* alleles have to be engineered for each mouse chromosome, and achieve meiotic recombination with a desired gene mutation (Zong, 2014). In order to significantly increase the number of genes that can be targeted mosaicly with this approach, the MADM construct has been targeted to all remaining mouse chromosomes, and this technology has now a coverage of more than 96% of the mouse genome (Contreras et al., 2020), even though there is still the need to interbreed a different *MADM* allele for every mouse gene/chromosome you want to study.

Another simpler approach is the mosaic mutant analysis with spatial and temporal control of recombination (MASTR) method (Lao et al., 2012), developed by the Alexandra Joyner laboratory. The *MASTR* allele (Figure 6B) is inducible by the Flp (or FlpE or FlpO) recombinase and subsequently results in the expression of the Cre-GFP fusion protein, which labels the nuclei of the Flp expressing cells. In this case, given that all GFP+ cells express Cre, the correlation between the expression of a reporter and a given floxed gene deletion is very high, even if the initial Flp or FlpOERT2 genetic pulse was very weak. In contrast to the MADM approach, with MASTR there is temporal control of recombination, and the allele is also compatible with any existent floxed allele. One of the disadvantages of the MASTR method, in comparison with the iSuRe-Cre method mentioned above, is the dependence on the very few available (and not tissue-specific) *FlpERT2* mouse lines, which are far less inducible than existent *CreERT2* lines. This results in the induction of very few clones of mutant cells in the tissue (Lao et al., 2012). A disadvantage of this method, in relation to the MADM method, is the absence of wildtype cells labeling, which prevents direct comparisons of mutant and wildtype cell phenotypes over a pulse-and-chase experiment. The exclusive nuclei labeling of the mutant cells expressing the *MASTR* allele also prevents the visualization of the cell shape, even though this allele can be combined with another cytoplasmic or membrane FP expressing Cre-reporter allele (Figure 2A). An important caveat noticed by the authors, is that the MASTR allele is leaky in the male germline, presumably due to skipping or alternative splicing of the transcription stop cassette. This results in the expression of GFP-Cre (even if very low) in the absence of Flp recombinase, which further complicates the experimental breedings and raise additional issues and the need of experimental controls to determine the genetic status of MASTR-reporter negative cells. With the MADM and MASTR approaches all mutant or wildtype cells will be labeled with a single FP, which prevents the distinction of different clones of wildtype or mutant cells if they occupy the same location. Therefore with both systems, the single-cell expansion or clonal analysis must rely on the assumption that the recombination events are very rare, and that there are no hot spots of recombination. Hot spots of recombination can occur when the expression of transgenic *Cre* or *Flp/FlpERT2* alleles occurs at very high levels in a small subset of cells, or when tamoxifen gets trapped or enriched in some tissue locations, which can significantly confound the assignment of single-cell-derived clones.

To overcome some of the caveats mentioned above, we recently developed an alternative genetic system, named as *Dual ifgMosaic*, to induce functional and multispectral combinatorial genetic mosaics (Pontes-Quero et al., 2017). This system is based on the Brainbow genetic mosaic system (Livet et al., 2007) mentioned above, and enables the complete correlation between the expression of a given FP and the genetic status of a cell. The functional Dual ifgMosaic lines are similar to the iChr-Mosaic and iMb-Mosaic lines described in Figures 5D,D’, but in this case some of the genetic elements contain downstream of the fluorescent proteins the viral 2A peptide and specific genes that either increase or decrease the function of a given signaling pathway in a cell-autonomous manner. In this way, the different fluorescent cells can be unambiguously identified as wildtype (express FP only) or mutant (co-express at equimolar levels FP and gene of interest). The Dual ifgMosaic technology also uses an open-source DNA engineering strategy that greatly simplifies the assembly of new functional genetic mosaic constructs. It allows the induction of one functional chromatin mosaic; expressing up to three different combinations of chromatin/nuclei localized FPs and genes and one functional membrane mosaic; expressing up to three different combinations of membrane localized FPs and genes (Figure 6C). By intercrossing mice expressing one functional mosaic allele, with other mice expressing a control (to increase clonal resolution) or functional mosaic allele, is possible to induce up to 15 different clones of cells in the same tissue, of the same animal, each of the clones expressing a given FP and gene, or combination of two genes and FPs. We have used the Dual ifgMosaic method to induce single and combinatorial Notch and VEGF signaling loss and gain-of-function genetic mosaics, which allowed us to obtain a deeper understanding of the short and long-term cell-autonomous roles of these pathways in vascular biology and how they functionally intersect at the single-cell level (Pontes-Quero et al., 2017, 2019). Given that ifgMosaic alleles rely on the easier to achieve, and Cre/CreERT2 dependent, intrachromosomal recombination events, they can be pulsed in any tissue at high rates. The multispectral and combinatorial dual (membrane and nucleus) cell labeling strategy, further facilitates the identification of single-cell-derived wildtype and mutant clones in the same tissue, and also enable the direct imaging of the wildtype and mutant cell’s chromatin and membrane. Like all technologies mentioned above, the ifgMosaic technology also has its caveats. The most important is that with the ifgMosaic system, a given genetic pathway loss-of-function is only partial and can only be achieved by expressing a protein having a dominant negative effect, such as a direct negative regulator of a pathway (i.e., *Oaz1* to suppress *Odc1* activity), a transcription factor that lost its activator domain (i.e., *DN-Maml1* to decrease NICD/*Maml1/Rbpj* complex signaling) or a receptor that lost its tyrosine kinase domain (i.e., *Vegfr2^TK−^*, to decrease VEGFR2 signaling). This requires *a priori* knowledge about a pathway’s biology or protein function. In addition, the overexpression of a protein having a dominant negative effect can lead to unpredictable biological consequences that differ from the endogenous pathway or gene function. In contrast to full genetic deletions or endogenous gene loss-of-function achieved with the *MADM* and *MASTR* alleles, with the *ifgMosaic* alleles a pathway or gene function is only partially suppressed, which sometimes can be more informative of a potential pharmacological compound effect, since pharmacological compounds also often do not completely ablate a given gene/protein/pathway function. With the advancement of CRISPR/Cas9 genetics and methods, is now possible to swap the genes in existent *ifgMosaic* alleles and mouse lines, by other genes of interest, which will greatly facilitate the generation of new *ifgMosaic* alleles.

In summary, even though reliable methods to induce and analyze functional genetic mosaics have been developed, all of them have their specific caveats and improvements in these technologies are needed to increase their ease of use, applicability, and reliability.

## Higher Resolution Lineage Barcoding With Cre or Cas9

The microscopy-based methods to follow clones or lineages of cells *in vivo* and *in situ* have their limitations. The first is the relatively low-throughput of microscopy-based imaging methods and analysis. The second is the relatively low number of FPs available, and their combinations, in order to distinguish the different clones of cells *in situ*. With the advancement of CRISPR/Cas9 genetics and DNA/RNA sequencing technologies, it is now possible to encode and decode cell lineages with much higher complexity and throughput by using bioinformatic methods. Most of the methods developed so far only allow *ex situ* lineage analysis, while a few enable *in situ* lineage barcode reading.

The first methods developed to generate single-cell or lineage DNA barcodes consisted in electroporating or infecting cells with a very complex mix of plasmids or viruses with known diverse sequences, so that only some of the many available DNA sequences would integrate in a given cell and generate a clone of cells sharing a given specific combination of sequences or barcode. This was possible after the development of high-scale DNA synthesis methods that enable the generation of a wide range of different sequences. When a group of transfected/infected cells has the same and unique genomic DNA barcode, they are related with a single lineage (Schepers et al., 2008). However, for most *in vivo* single-cell lineage studies the barcoding method needs to be more complex, controllable in time and not dependent on the exogenous delivery of genetic material.

**Figure 7:**
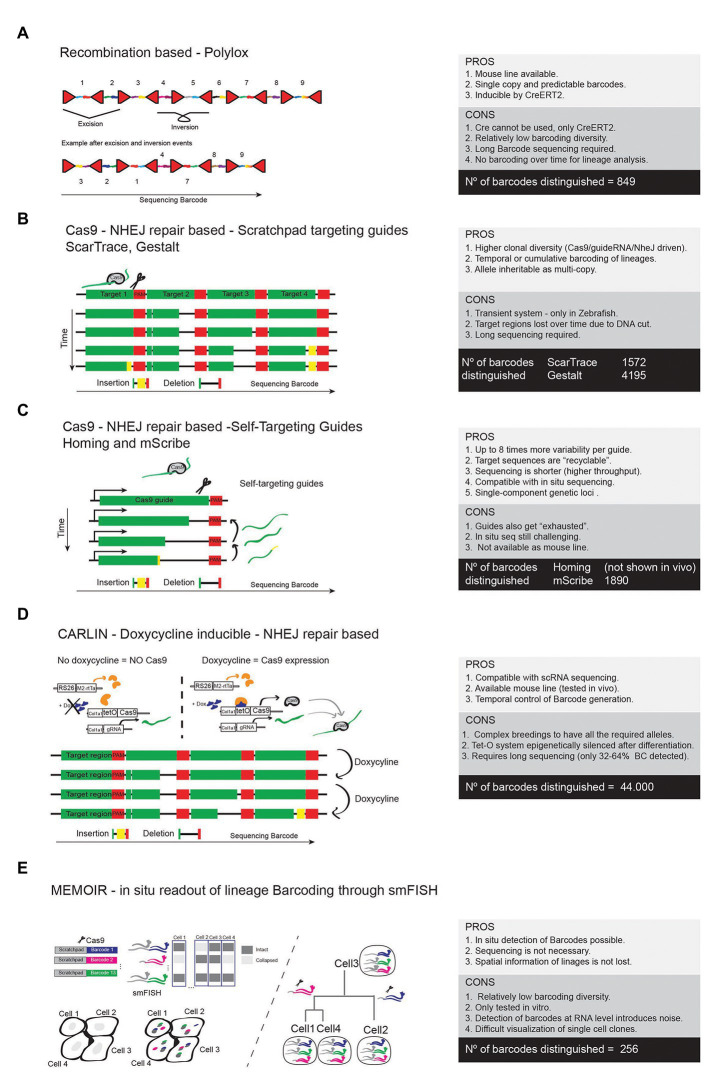
High resolution lineage barcoding with Cre or Cas9. **(A)** Scheme of the allele and main features of the Polylox barcoding method. **(B)** Scheme representing the Cas9/NHEJ-based barcoding methods that rely on guides targeting a pre-designed genomic scratchpad to generate evolving gene editing events and barcoding diversity. **(C)** Methods using self-targeting guides to generate barcoding diversity. **(D)** The CARLIN mouse line is based on the method shown in **B**, but is inducible by doxycycline, allowing temporal control of the genetic barcoding. **(E)** The MEMOIR barcoding approach uses a guide RNA-targeted scratchpad linked to different genetic barcodes. Different cells will accumulate a different combination of edited (collapsed) barcodes that are detectable by smFISH and microscopy.

One of the first systems developed to generate inducible barcodes within the genome of mouse cells was inspired by some of the recombinase-based combinatorial mosaic strategies described before. The *R26-Polylox* mouse line (Pei et al., 2017) enables Cre-recombinase-driven DNA barcoding by providing a 2.1 kb DNA cassette containing 10 *LoxP* sites in forward and reverse orientations. This results in highly diverse and stochastic recombination events (Figure 7A). Given the large size of the DNA cassette, targeted sequencing of a pre-amplified PCR product derived from single cell DNA must be employed. In practical terms the authors were able to detect only 849 different barcodes (from 1.866.868 theoretically possible) in up to 52% of sequenced cells (Pei et al., 2017). This is because the excision DNA deletion events are intrinsically favored over DNA inversion/flipping events. With *R26-Polylox*, Cre expression needs to be time restricted in order to avoid the collapse of the Polylox DNA cassette which results in a significant reduction in barcoding diversity (Peikon et al., 2014). Another caveat of this technology is that the sequencing of the relatively long barcode (up to 2.1 kb) requires long-read sequencing given the unpredictability of the incomplete recombination event, which is more expensive and has a lower throughput.

With the advancements in the CRIPR-Cas9 genome editing technology, several laboratories moved to inducible sgRNA/Cas9 genome editing systems in order to generate unique DNA barcodes in a cell’s genome by exploring the ease of targeting any DNA sequence with sgRNA/Cas9 and the randomness and diversity of the NHEJ repairing event. In contrast to the Polylox technology, the Cas9-based systems have the capacity to generate more barcoding diversity and enable cumulative barcoding, which is essential to understand how lineages of cells branch or develop over time (Figures 7B,C). All Cas9-based technologies generate variability due to random NHEJ repair mutations (deletions or insertions) that Cas9 induces after targeting and cutting a DNA sequence.

The first group of Cas9-based barcoding technologies is represented by ScarTrace (Alemany et al., 2018), Gestalt (Raj et al., 2018), and MEMOIR (Frieda et al., 2017). They all contain many repeats of a single target sequence that will be recognized and mutated by Cas9 when guided by the corresponding target RNA guide. Their barcode generation relies on the different NHEJ repair events that will occur in each targeted sequence over time (Figure 7B). These variable gene editing events can later be detected by targeted single cell RNAseq or DNAseq. With these methods, the barcoding diversity is limited to the number of repeats of the target sequence, because once one of these sequences is mutated it cannot be recognized and mutated again. In addition, when multiple sequences are targeted simultaneously by Cas9, the target array can be deleted, which results in a reduction of barcoding diversity. Nonetheless, the detected barcoding diversity was in the range from 1,572 to 4,195. ScarTrace and Gestalt were only successfully used in Zebrafish and they rely on direct delivery/injection of Cas9 and target sequences, which is not easy to translate into mouse models.

The second group of Cas9-based barcoding technologies tries to tackle the problem of reduction of available target sequences over time, and is represented here by Homing-CRISPR (Kalhor et al., 2017) and mSCRIBE (Perli et al., 2016). The basic differentiation principle of this technology is that the RNA guide is coded by the target sequence itself (Figure 7C). This means that mutation after mutation a different RNA guide is produced and changes in the barcode are accumulated one after the other, which allows for about eight times more variability per guide than the previous technologies. For this reason, this approach does not require a long array of target sequences and therefore a single-component genomic locus can be generated. The shorter sequencing required also turns the technology more cost-effective. However, even though the target sites last longer with Homing-CRISPR and mSCRIBE, the guides and target sequences tend to evolve too fast and after several cycles of mutations they also get “exhausted,” limiting the diversity generated by each coding element. Indeed, the detected barcode diversity is in the range of the previous technologies (Figure 7C).

Recently a mouse line, named as CARLIN (Bowling et al., 2020), was generated using constructs similar to the GESTALT approach. This mouse enables for the first time simultaneous readout of lineage histories and scRNAseq-based gene expression analysis in mice. The CARLIN mouse requires the combination of three independent alleles targeted to the Col1a1 or *Rosa26* locus (Figure 7D). One allele drives the Tet inducible expression of Cas9, the other Dox inducible *rtTA*, and a third construct expresses the Cas9 RNA guide and the targetable scratchpad to generate barcodes. CARLIN barcoding diversity is in the range of 44.000. However, CARLIN is based on the Tet-O system, which is known to be silenced epigenetically once cells differentiate (Godecke et al., 2017). In adult tissues, 7 days of continuous induction with Dox, reached editing ratios close to 0% in brain, heart, and muscle and 31–88% in other organs. In addition, the *CARLIN* Cas9 target array allele undergoes stochastic and unpredictable sgRNA Cas9 editing events that require special bioinformatic analysis. Due to the need for targeted amplification of the long CARLIN array, it could only be detected in 32–64% of single cell transcriptomes. An additional caveat of the CARLIN mouse is its reliance on the combination of three alleles, which need to be combined with at least three more alleles (two floxed alleles and one Cre allele) to induce barcoding of mutant cells, something that may take a long time to achieve.

With most of the barcoding technologies above, the cell *in situ* spatial information is lost, since cells need to be separated from the tissue and sequenced in order to detect the barcode. To solve this problem, some laboratories started to couple methods of cellular barcoding with methods of multiplexed and sequential single-molecule fluorescent *in situ* hybridization (seqFISH or smFISH). One example is the MEMOIR technology (Frieda et al., 2017). MEMOIR uses the same barcoding principle to the first groups of tools presented above (GESTALT and SCARTRACE). MEMOIR barcode is formed by the link between barcode sequences and repeated target sequences (scratchpads) that are sequentially mutated/collapsed by Cas9 activation. In this way, the *in situ* identification is based on the presence (if not collapsed/mutated) or absence (if collapsed/mutated) of the associated scratchpad in a binary way (Figure 7E). Identification of these changes relies on seqFISH, where one probe binds and recognizes one barcode and another probe binds to the scratchpad linked to that barcode. If the scratchpad associated to that barcode has collapsed the cell will only have the signal coming from the barcode associated to the FISH probe, but not to the scratchpad probe (Figure 7E). Since each cell has up to 13 expressed barcode-scratchpad tandems integrated as multicopy, the final read-out will define the number and location of intact scratchpads, which at the end serves to generate barcoding diversity in each cell. Simplifying, if one cell has intact scratchpads associated with barcodes 1, 3, 5, and 13, we can associate that cell with other cells having the same pattern. We can also detect lineages by the progressive loss of scratchpads. This allows the readout of cell lineages *in situ*, which give us spatial and temporal information of cell linage evolution. However, this technique also has some caveats. In MEMOIR, the seqFISH technology was demonstrated in mouse ES cells cultured *in vitro*, but will be far more difficult to implement this *in vivo* given the number of cells and size of tissues. seqFISH is also very costly and time consuming, and few labs can afford to do it in a routine manner. The seqFISH signals are tiny dots within the cells and do not label the entire cell like a fluorescent reporter, which turns difficult the visualization of single-cell clones in tissues. RNA expression is also known to fluctuate immensely from cell to cell, which introduces noise and false negatives in the clonal analysis. On top of this, this approach has been proven to be able to detect only up to 256 different barcodes, which may not be enough to follow-up complete cell linages *in vivo*. The power and throughput of seqFISH barcoding readout and analysis *in vivo* remains low, when compared to *ex situ* single cell sequencing (Frieda et al., 2017), but new developments in 3D seqFISH and microscopy images processing may improve its capacity and throughput (Wang et al., 2018a; Eng et al., 2019; Xia et al., 2019).

The methods presented above are more a proof-of-concept, than already established and easy-to-use technologies. They are all relatively recent, and need improvements, but they were all published in high profile journals given their enormous potential to allow the reconstruction of lineage relationships of individual cells *in situ* and *ex situ*. There are still several things to optimize in these barcoding technologies, particularly for their efficient use in mouse models and conditional genetic approaches. It is essential to have temporal and spatial control of barcoding, not just of its start, but also of its progression over time. It is also important to increase the diversity of barcoding and reduce its unpredictability in order to facilitate the downstream bioinformatic analysis. The lineage or single-cell barcoding also needs to be combined with reliable conditional gene loss-of-function methods and both need to be detectable by *in situ* or *ex situ* microscopy and sequencing technologies.

## Reporters of Cardiovascular Cell Dynamics and Signaling Activity

**Figure 8:**
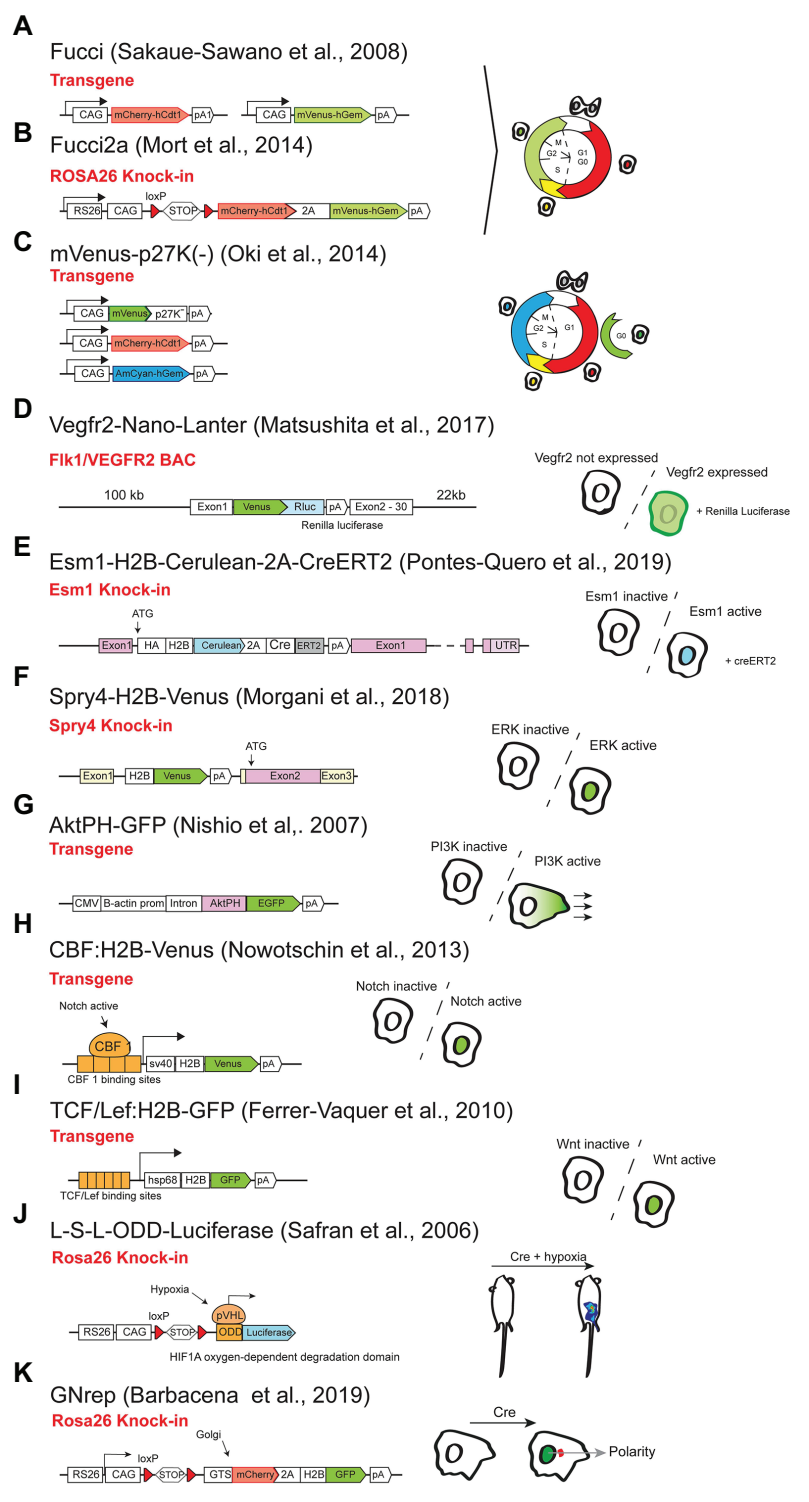
Reporters of cardiovascular cell dynamics and signalling. Representation of the different reporter alleles on the left. On the right is possible to see the type of cell labelling achieved according to the stage of the cell-cycle or cell signalling/polarity activity.

The use of cellular or signaling activity reporters has been very important to visualize the regulation of genes or pathways during tissue development, homeostasis and in disease. One of the most important cellular processes is the cell cycle progression. Being able to track and follow the cell-cycle status in individual cells can be very useful to understand the role of genes in the regulation of the cell-cycle. *Fucci*, is the name of the first method and mouse line to be produced in order to track the different stages of the cell-cycle (Sakaue-Sawano et al., 2008). This method consisted in inducing the strong and continuous expression of two different tagged FPs that are subjected to post-translational regulation depending on the cell-cycle stage. One of the proteins contains the *hCdt1* (*human Chromatin Licensing And DNA Replication Factor 1*) domain and is fused with the fluorescent mCherry, that accumulates in the G0 or G1 phases of the cycle, and is actively degraded in the other stages. The other reporter contains the *hGem (human Geminin)* domain fused with the fluorescent Venus, which accumulates only in the S/G2/M phases of the cycle (Sakaue-Sawano et al., 2008; Mort et al., 2014). By quantifying the relative levels of the two proteins is possible to determine cell-cycle stages and transitions. The first *Fucci* mouse line consisted in the use of two separate transgenes (randomly integrated) driving expression of the two proteins (Figure 8A; Sakaue-Sawano et al., 2008), which is not ideal and is sensitive to epigenetic variegation. The second *Fucci* mouse line has a single construct knocked-in the *Rosa26* locus and uses the viral 2A peptide which allows equimolar expression of the two proteins, providing a more reliable readout of the cell-cycle (Figure 8B; Mort et al., 2014). Moreover, this *Fucci2a* line is inducible by Cre, which permits tissue specific labeling. In the cardiovascular field, the FUCCI technology has been used to understand the regulation of cell-cycle during vascular network formation (Yasuda et al., 2016); or to study cardiomyocyte cell cycle progression or arrest in the context of myocardial infarction and early postnatal development (Alvarez et al., 2019); or hemogenic endothelium and hematopoietic development (Zape et al., 2017). The line is also available in its zebrafish endothelial-specific version (Fukuhara et al., 2014). A more recent method (FUCCI4) uses four orthogonal fluorescent indicators that together resolve all cell-cycle phases, to provide even more cell-cycle resolution (Bajar et al., 2016). However, a mouse line carrying FUCCI4 still does not exist.

The above mentioned lines can also be used to isolate cells at different stages of the cell-cycle. The main caveats of the FUCCI technology is the lack of distinction between cells in G0 (Ki67−) and G1 (Ki67+) phases of the cell-cycle and the lack of fluorescence in cells after the M-phase or initial G1 phase of the cycle. A solution to this may be the expression of p27-tagged fusion proteins that seem to label G0 cells (Figure 8C; Oki et al., 2014). However, this does not seem yet a universal solution. Additionally, it would be ideal to combine all these different cell-cycle markers in an all-in-one genetic tool or mouse line.

The other subset of mouse lines is useful as reporters of activity of signaling pathways important for the biology of cardiovascular cells. This is the case for reporters of activity for the VEGF, Notch, Wnt, ERK, and PI3K signaling pathways. In the case of the VEGF pathway, there are several mouse lines that report the expression of the VEGF receptors or ligands *Vegfr2-GFP* (Ishitobi et al., 2010), *Vegfr3-YFP* (Calvo et al., 2011), *Vegfr2-nano-LANTER* (Figure 8D; Matsushita et al., 2017), but very few reporting VEGF signaling activation. One of the lines that can be used for this purpose is the *Gt(Esm1)^tm1(HA-H2B-Cerulean-2A-iCreERT2)^* line, expressing H2B-Cerulean in cells with expression of *Esm1* (Figure 8E), a gene that is upregulated only in ECs with very high VEGF signaling, such as retina endothelial tip cells (Rocha et al., 2014; Pontes-Quero et al., 2019). It would be very useful to develop another VEGF signaling reporter, more sensitive to lower or oscillatory levels of VEGF signaling.

Given the strong regulation of ERK signaling by VEGF, and the central importance of this signaling cascade for cell biology, mouse lines reporting its activity can be very useful. In *C. elegans* and Zebrafish a biosensor was successfully used (de la Cova et al., 2017; Mayr et al., 2018) and accurately reports ERK signaling activity. A knock-in of the mouse gene *Spry4*, which is a very sensitive and specific target of ERK activity (Figure 8F; Morgani et al., 2018) can also be used, however the high stability of H2B-Venus may be an issue for reporting ERK signaling dynamics. These reporter-expressing approaches are particularly suitable for live imaging experiments or to increase the sensitivity of ERK signaling detection, given that an anti-P-ERK antibody (D13.14.4E – cell signal) already enables the detection of cells with high ERK activation in fixed tissues.

Like the ERK pathway, the PI3K pathway also has a major function in vascular biology, and the *AktPH-GFP* transgenic allele expresses a probe for PtdIns(3,4,5)P_3_, which can be used to monitor the activity of this pathway (Figure 8G; Nishio et al., 2007), even though it has not been widely used.

For Notch signaling, there are several mouse reporter lines, but none accurately reports the endogenous Notch signaling activity in cardiovascular cells. The *CBF:H2B-Venus* line (Nowotschin et al., 2013) displays high and bright nuclei Venus signal in a subset of tissues with *Rbpj/Notch* activity (Figure 8H), such as blood vessels (Mack et al., 2017; Salguero-Jiménez et al., 2018; Diéguez-Hurtado et al., 2019), but intense fluorescent signals are still present for several days after deletion of *Rbpj* or after blocking Notch signaling with DAPT or anti-Dll4 (unpublished data). The *TNR* line (Duncan et al., 2005) has also shown to report Notch activity in some vascular cells (Hellstrom et al., 2007), but its transgene expression pattern is highly variable and does not reflect the endogenous Notch activity in most cardiovascular cells. Several BAC or knock-in reporter lines for the Notch target genes *Hes1/Hes5/Hey1* were generated (Heintz, 2004), with stable or unstable FPs, but they also have the problem of under or over-reporting Notch activity (Imayoshi et al., 2013). Elegant Hes expression reporters using super-unstable luciferase reporters have been successfully used to image oscillations in Notch activity, however, *Hes1/5* genes, unlike *Hey1/2*, are not strong targets of Notch signaling in endothelial cells. In addition the *Hes/Hey* genes are not only regulated by Notch, but also BMP signaling (Itoh et al., 2004). So far the best method to detect Notch receptor activation in vascular cells, is to stain for the active N1ICD protein with the antibody Val1744 (Cell signaling), even though the signal is very weak and often needs tyramide-based signal amplification for adequate detection.

Another relevant pathway in the cardiovascular field is the Wnt pathway. This signaling pathway is an important regulator of cell proliferation, differentiation, and cardiovascular tissue patterning (Foulquier et al., 2018). The *TCF/Lef:H2B-GFP* mouse allele contains six copies of the TCF/Lef Wnt signaling responsive element together with a minimal promoter and a fluorescent reporter, which enables the visualization and isolation of cells with high Wnt activity (Figure 8I; Ferrer-Vaquer et al., 2010; Wang et al., 2018b).

Hypoxia and oxygen sensors or reporters are also very useful genetic tools to understand cardiovascular biology and the *ODD-Luc* mouse (Safran et al., 2006), uses the Oxygen-Dependent Degradation Domain (ODD) of the *Hif1a* gene in order to express luciferase protein (Figure 8J). A Cas9 editing or conversion of this luciferase into a FP could increase its utility.

Another process that is commonly analyzed in most cardiovascular studies is cell migration. This cellular behavior is not only necessary for development, but it also plays an important role in disease, regeneration, and tissue homeostasis. One of the main features that define cell migration is cell polarity, and being able to *in vivo* label cellular orientation is necessary to predict its migratory direction. The new *GNrep* mouse line (Barbacena et al., 2019) is able to do this by double labeling the nuclei and the Golgi complexes of the cells, which provides a read-out of the cellular migratory axis. Since this line is inducible by Cre, it enables tissue-specific cell polarity labeling and analysis (Figure 8K).

A problem that might arise with the use of fluorescent reporter lines, especially the highly expressed ones, is toxicity. Some fluorescent proteins like GFP have been extensively tested in mice, and are known to be generally safe. However, some red fluorescent proteins have been shown to be toxic in mice. For example, there is no mouse line expressing the native dsRed protein due to toxicity. In fact, the oligomerization to a tetramere and its long maturation time have been proposed as an explanation to its toxicity *in vivo* (Shaner et al., 2005). To solve this problem, new red fluorescent proteins derived from dsRed, like Cherry and tdTomato, have been generated and there are today numerous mouse lines expressing these fluorescent proteins constitutively without any reported abnormal phenotype (Muzumdar et al., 2007). Independently of the native protein being more or less toxic, location of the protein within the cell and levels of expression are also very important factors to consider and control in genetic experiments using new reporter lines. Therefore, *in vitro* toxicity reported in transient overexpression assays, in which each cell is transfected with numerous copies of a plasmid, cannot be used to predict toxicity of unicopy expression of fluorescent reporters in mouse lines *in vivo* (Detrait et al., 2002). In addition, expression of tagged fluorescent and non-fluorescent proteins may also interfere with the endogenous reported protein function (Hammond et al., 2010). Nonetheless, despite the need for adequate controls, reporter lines are valuable tools to better understand the dynamics of cardiovascular related processes and signaling pathways.

## Conclusion and Outlook

The study of vascular biology has progressed immensely with the development of new genetic tools and mouse models. From the first endothelial/pericyte/SMC-specific transgenic lines expressing a single FP or Cre, to the use of dual recombinase genetics, multispectral combinatorial labeling, cellular barcoding or scRNAseq to deconstruct single-cell transcriptomes and lineages. The field has now access to an immense diversity of alleles and methodologies. However, animal experiments are costly, and few laboratories can afford to maintain a large stock of animals and mouse lines or alleles. It is therefore essential to make informed decisions on which genetic technology works better and is key to answer a given biological question.

Looking forward it will be very important not only to develop new technologies but also optimize existing technologies. Most of the technologies mentioned in this review have inherent caveats that limit their simplicity of use or direct broad application. Many of these caveats could be overcome with significantly more investment. It is nonetheless frustrating the lack of general funding and investment to refine existing technology or to develop new genetic tools. Most of the new alleles and technologies are being developed by individual laboratories as side-projects, not as grant-funded projects, since it is far easier to get funding to work on cancer or cardiovascular biology and its impact in disease than to “just” establish a new genetic tool or method, even if it may have a much broader impact on the way a field studies and understands cancer or cardiovascular biology. A clear example of this imbalance in disease versus basic/technology-oriented funding is the limited funding devoted over time to the seminal research that allowed the iPSC or CRISPR/Cas9-based technologies to be established and significantly boost biomedical research.

With the new wave of single-cell technologies and CRISPR/Cas9-based cellular barcoding and gene targeting methods, we can now target genomic sequences, record gene editing events, and decode cellular histories and single-cell lineages with unprecedented throughput and resolution *ex situ*. The development of FISH and other multiplexed *in situ* sequencing technologies are also significantly expanding our capacity to detect stochastic cellular barcodes *in situ*. The combination of these technologies will certainly push the limits of what is possible to do with mouse genetics and the type of biological questions that can be answered. They may also allow us one day to deconstruct the entire normal or mutant tissue development or disease dynamics by analyzing only its endpoint. This will require the coordinated efforts of several laboratories with different skills in mouse *in vivo* gene-targeting/editing, microscopy, FISH, scRNAseq, and bioinformatic analysis. The continuous development and refinement of genetic methods of broad interest will certainly set the foundation to increase the efficiency and output of biomedical research.

## Author Contributions

MF-C and SM did literature search and wrote some sections. IG-G and RB wrote most of the manuscript and assembled the figures. All authors contributed to the article and approved the submitted version.

### Conflict of Interest

The authors declare that the research was conducted in the absence of any commercial or financial relationships that could be construed as a potential conflict of interest.
